# *PDX1* and *MC4R* genetic polymorphisms are associated with type 2 diabetes mellitus risk in the Chinese Han population

**DOI:** 10.1186/s12920-021-01037-3

**Published:** 2021-10-25

**Authors:** Ning Wang, Rui Tong, Jing Xu, Yanni Tian, Juan Pan, Jiaqi Cui, Huan Chen, Yanqi Peng, Sijia Fei, Shujun Yang, Lu Wang, Juanchuan Yao, Wei Cui

**Affiliations:** 1 Department of Endocrinology and Second Department of Geriatrics, The First Affiliated Hospital of Xi’an Jiaotong University, 277 West Yanta Road, Xi’an, 710061 Shaanxi China; 2 Department of Oncology, East Branch of the First Affiliated Hospital of Xi’an Jiaotong University, Xi’an, 710089 Shaanxi China; 3 Department of Endocrinology, Xianyang Central Hospital, Xianyang, 712000 Shaanxi China

**Keywords:** Type 2 diabetes mellitus, *PDX1*, *MC4R*, Polymorphism, Susceptibility

## Abstract

**Background:**

Diabetes mellitus (DM) is a complex metabolic disease that is caused by a complex interplay between genetic and environmental factors. This research aimed to investigate the association of genetic polymorphisms in *PDX1* and *MC4R* with T2DM risk.

**Methods:**

The genotypes of 10 selected SNPs in *PDX1* and *MC4R* were identified using the Agena MassARRAY platform. We utilized odds ratio (OR) and 95% confidence intervals (CIs) to assess the correlation between genetic polymorphisms and T2DM risk.

**Results:**

We found that *PDX1*-rs9581943 decreased susceptibility to T2DM among in a Chinese Han population (OR = 0.76, *p* = 0.045). We also found that selected genetic polymorphisms in *PDX1* and *MC4R* could modify the risk of T2DM, which might also be influenced by age, sex, BMI, smoking status, and drinking status (*p* < 0.05).

**Conclusions:**

We concluded that *PDX1* and *MC4R* genetic variants were significantly associated with T2DM risk in a Chinese Han population. These single polymorphic markers may be considered to be new targets in the assessment and prevention of T2DM among Chinese Han people.

**Supplementary Information:**

The online version contains supplementary material available at 10.1186/s12920-021-01037-3.

## Background

Diabetes mellitus (DM) is a metabolic disease characterized by the presence of chronic hyperglycemia, which results from either weakened insulin secretion or insulin action or both [1]. The global prevalence of diabetes reached 9.3% (463 million) in 2019, and it is expected to increase to 10.9% (700 million) by 2045 [2]. China has the highest number of adults with diabetes, approximatedly116 million, ranking first in diabetes prevalence worldwide [2]. Type 2 diabetes mellitus (T2DM) accounts for nearly 90% of the total diabetes patients. There are multiple reasons for the incidence of T2DM including aging, sedentary lifestyles and genetic factors [3]. It has been reported that subjects withT2DM-affected siblings have a two- to three fold increased risk of developing T2DM compared with the general population [4]. Having one parent with diabetes increases the risk of developing T2DM by 30–40%, and having two parents with diabetes increases the risk to 70% [5]. Furthermore, some research reported that genetic polymorphisms in candidate genes could influence the formation and course of T2DM [6, 7].

Pancreatic and duodenal homeobox-1 (*PDX1*) modulates pancreas development and β-cell function. The *PDX1* gene encodes a protein of 283 amino acids in humans. It also regulates many genes, such as those encoding insulin and glucokinase (GK), involved in maintaining the function of β-cells. In adults, *PDX1* is highly expressed in β-cells, where it is required for efficient insulin gene transcription [8]. Indeed, *PDX1* has been proposed to be an oncogene, since its overexpression increased pancreatic cancer cell proliferation, invasion, and growth in humans [9]. Gurevich et al. also illustrated that *PDX1* was upregulated in neuroendocrine tumors, including pancreatic ductal and acinar cell tumors and gastric signet ring cell carcinomas [10]. It has previously been noted that *PDX1* deficiency inhibits the development of pancreatic buds, leading to extreme hyperglycemia [11]. These findings demonstrated that *PDX1* plays a pivotal role in the development of pancreas-related disease. However, no literature supports the effect of *PDX1* polymorphisms on T2DM.

Melanocortin receptor 4 (*MC4R*) belongs to class A of G protein-coupled receptors and is a member of the melanocortin receptor family [12]. *MC4R* can control energy homeostasis, sympathetic nervous system activity, and blood pressure in rodents and humans [13]. For instance, *MC4R* knockdown mice were severely obese and the loss of one *MC4R* allele resulted in an intermediate obesity phenotype [14]. Greenfield et al. demonstrated reduction in blood pressure and circulating catecholamine levels in humans with *MC4R* deficiency [15]. In addition, previous research has established that *MC4R* deletion or mutation results in obesity, hyperphagia, and insulin resistance [16]. These observations highlight a potential role for *MC4R* in obesity-related diseases. In addition, obesity is believed to be an independent risk factor for T2DM [17]. Based on the above information, we hypothesized that *MC4R* may be involved in the occurrence of T2DM.

Therefore, we mainly examined the role of *PDX1* and *MC4R* genetic polymorphisms in T2DM development in a Chinese population. We identified four polymorphisms in *PDX1* (rs11619319, rs2293941, rs9581943 and rs7981781) and six polymorphisms in *MC4R* (rs6567160, rs663129, rs17782313, rs12969709, rs11663816, and rs12970134) to investigate the correlations between genetic polymorphisms and T2DM susceptibility. The current study will provide new targets for the early assessment and prevention of T2DM.

## Methods

### Study population

A total of 500 T2DM patients and 501 healthy controls were enrolled from the First Affiliated Hospital of Xi’an Jiaotong University in the present study. All patients were diagnosed with T2DM based on fasting plasma glucose ≥ 7.0 mmol/L or postprandial plasma glucose ≥ 11.1 mmol/L or HbA1c ≥ 6.5% [18]. Patients with type 1 diabetes mellitus; gestational diabetes; acute or chronic diseases of the liver, kidney, or heart; other endocrine diseases; inflammatory diseases; or malignant tumors were excluded. The inclusion criteria for controls were no history of diabetes, metabolic disorders or severe diseases. The demographic and clinical characteristics of all subjects, including age, sex, smoking status, drinking status, complications, and body mass index (BMI), were collected from medical records and questionnaires.

This research received approval from the Ethics Committee of the First Affiliated Hospital of Xi’an Jiaotong University, and conformed to the Declaration of Helsinki. Informed consent was acquired from each participant at recruitment after fully describing our research to them.

### SNP genotyping

We selected four SNPs in *PDX1* and six SNPs in *MC4R* and all SNPs had minor allele frequencies (MAFs) ≥ 5% in the 1000 Genomes Chinese Han Beijing population. Peripheral blood samples (5 mL) were collected from each subject, and genomic DNA was extracted using the GoldMag whole-blood DNA purification kit (GoldMag Co.Ltd., Xi’an, China) following the manufacturer’s protocol. Genotyping of *PDX1* and *MC4R* polymorphisms was performed by the Agena MassARRAY platform (Agena Bioscience, San Diego, CA, USA). Moreover, Agena Typer 4.0 software was used to analyze and manage data.

### Gene expression analysis

We performed *PDX1* and *MC4R* mRNA expression analysis with blood samples from 100 unrelated Chinese Han individuals. Total RNA was isolated from peripheral blood using a Qiagen kit (Qiagen) according to the manufacturer’s instructions. RNA was reverse transcribed to synthesize first-strand cDNA using the PrimeScript_-_1st strand cDNA Synthesis Kit (Takara Bio, Shiga, Japan), as described by the manufacturers. The mRNA expression of the *PDX1* and *MC4R* genes and the internal control *GAPDH* were assessed using quantitative real-time PCR (ABI PRISM 7500 Real-Time PCR System; Applied Biosystems). The relative mRNA expression was calculated by the 2^−Δ(ΔCt)^ comparative method and normalized to GAPDH expression.

The primer sequences for the mRNA expression of *PDX1*, *MC4R* and *GAPDH* are shown in Additional file 1: Table S1. Amplification was performed in a reaction mixture containing 10 pM each primer, 10 μl SYBR Green/High ROX (Amplicon), 7 μl nuclease-free water, and 2 μl cDNA solution. Experiments were performed in triplicate.

### Statistical analysis

Statistical differences in demographic characteristics of the participants were assessed using the χ^2^ test and Student′s t-test. Hardy–Weinberg equilibrium (HWE) of each SNP among controls was evaluated using the χ^2^ test. The association of the selected SNPs with T2DM susceptibility was examined by odds ratio (ORs) and 95% confidence intervals (CIs) by logistic regression analysis in multiple inheritance models and different subgroups (age, sex, smoking, drinking and BMI). The potential functions of the selected SNPs were forecasted using HaploReg v4.1 (https://pubs.broadinstitute.org/mammals/haploreg/haploreg.php). Haploview software and PLINK software were used for Haploview analysis and linkage disequilibrium [19, 20]. The mRNA expression was analyzed using Student’s *t*-test in the case and control groups. The effects of the polymorphisms on mRNA expression were examined by one-way analysis of variance (ANOVA). A *p* value < 0.05 was considered statistically significant.

## Results

### Characteristics of the study population

As presented in Table 1, there were 500 T2DM patients (358 men and 142 women) and 501 healthy controls (358 men and 143 women) in this study. The average ages were 59.87 ± 12.87 years for cases and 59.85 ± 9.34 years for controls. There were no significant differences in age (*p* = 0.973) or sex (*p* = 0.960) between the case and control groups. In addition, significant differences were observed in total cholesterol (*p* < 0.001), low-density lipoprotein cholesterol (LDL-C, *p* = 0.012), high-density lipoprotein cholesterol (HDL-C, *p* = 0.024), fasting blood glucose (*p* < 0.001) and urea (*p* < 0.001) between the two groups.

**Table 1:** Characteristics of the study population

Characteristics	Cases (n = 500)	Controls (n = 501)	*p*
Age, years			
Mean ± SD (years)	59.87 ± 12.87	59.85 ± 9.34	0.973^a^
> 60	240 (48%)	268 (54%)	
≤ 60	260 (52%)	233 (46%)	
Sex			0.960^b^
Male	358 (72%)	358 (71%)	
Female	142 (28%)	143 (29%)	
Smoking			
Yes	219 (44%)	98 (20%)	
No	280 (56%)	164 (33%)	
Absence	1	239 (47%)	
Drinking			
Yes	109 (22%)	103 (21%)	
No	385 (77%)	140 (28%)	
Absence	6 (1%)	258 (51%)	
BMI			
≤ 24	203 (41%)	130 (26%)	
> 24	239 (48%)	188 (38%)	
Absence	58 (11%)	183 (36%)	
Complication			
One	107 (21%)		
Multiple	337 (67%)		
Absence	56 (12%)		
Total cholesterol (mmol/L)	4.19 ± 2.01	4.93 ± 4.00	**< 0.001**^**a**^
LDL-C (mmol/L)	2.45 ± 0.90	2.62 ± 0.76	**0.012**^**a**^
HDL-C (mmol/L)	1.05 ± 0.72	1.15 ± 0.55	**0.024**^**a**^
Fasting blood glucose	7.35 ± 3.40	6.05 ± 1.60	**< 0.001**^**a**^
Triglyceride	1.91 ± 1.91	1.74 ± 0.10	0.088
GFR(ml/min)	96.62 ± 22.22	96.01 ± 19.78	0.710
Urea	6.52 ± 3.26	5.42 ± 2.78	**< 0.001**^**a**^
Creatinine	71.20 ± 52.66	68.74 ± 12.87	0.371

Bold indicates a statistically significant (*p* < 0.05).

*SD* standard deviation, *BMI* body mass index, *HDL-C* high-density lipoprotein cholesterol, *LDL-C* low-density lipoprotein cholesterol

*p*^a^ value obtained from an independent sample *t*-test

*p*^b^ value obtained from Pearson's χ^2^ test

### T2DM risk assessment

Four candidate SNPs in *PDX1* (rs11619319, rs2293941, rs9581943, and rs7981781) and six SNPs in *MC4R* (rs6567160, rs663129, rs17782313, rs12969709, rs11663816, and rs12970134) were successfully genotyped, as shown in Additional file 1: Table S2. Deviation from HWE was assessed in controls and all candidate SNPs reached the expected *p* values (*p* > 0.05). There were no significant associations between allele frequencies of any SNP and susceptibility to T2DM (*p* > 0.05).

**Table 2:** Relationships of polymorphisms in *PDX1* and *MC4R* and T2DM risk

Gene	SNP	Model	Genotype	Without adjustment		With adjustment	
				OR (95% CI)	*p*^a^	OR (95% CI)	*p*^b^
*PDX1*	rs11619319	Codominant	AA	1.00		1.00	
			GG	1.09 (0.76–1.56)	0.629	1.09 (0.76–1.56)	0.629
			GA	0.90 (0.68–1.20)	0.471	0.90 (0.68–1.20)	0.471
		Dominant	AA	1.00		1.00	
			GG-GA	0.95 (0.73–1.25)	0.715	0.95 (0.73–1.25)	0.717
		Recessive	GA-AA	1.00		1.00	
			GG	1.17 (0.85–1.59)	0.333	1.17 (0.85–1.59)	0.334
		Additive	–	1.03 (0.86–1.23)	0.755	1.03 (0.86–1.23)	0.756
*PDX1*	rs2293941	Codominant	GG	1.00		1.00	
			AA	1.09 (0.76–1.56)	0.646	1.09 (0.76–1.56)	0.646
			AG	0.89 (0.67–1.18)	0.425	0.89 (0.67–1.18)	0.426
		Dominant	GG	1.00		1.00	
			AA-AG	0.94 (0.72–1.23)	0.666	0.94 (0.72–1.23)	0.667
		Recessive	AG-GG	1.00		1.00	
			AA	1.17 (0.85–1.60)	0.332	1.17 (0.85–1.60)	0.333
		Additive	–	1.02 (0.86–1.22)	0.791	1.02 (0.86–1.22)	0.792
*PDX1*	rs9581943	Codominant	GG	1.00		1.00	
			AA	0.96 (0.65–1.42)	0.844	0.96 (0.65–1.41)	0.842
			AG	0.76 (0.58–0.99)	**0.046**	0.76 (0.58–0.99)	**0.045**
		Dominant	GG	1.00		1.00	
			AA-AG	0.80 (0.63–1.04)	0.090	0.80 (0.62–1.04)	0.090
		Recessive	AG-GG	1.00		1.00	
			AA	1.11 (0.77–1.59)	0.574	1.11 (0.77–1.59)	0.574
		Additive	–	0.92 (0.77–1.10)	0.353	0.92 (0.77–1.10)	0.354
*PDX1*	rs7981781	Codominant	GG	1.00		1.00	
			AA	1.08 (0.75–1.54)	0.681	1.08 (0.75–1.54)	0.681
			AG	0.85 (0.64–1.13)	0.263	0.85 (0.64–1.13)	0.263
		Dominant	GG	1.00		1.00	
			AA-AG	0.91 (0.70–1.19)	0.486	0.91 (0.70–1.19)	0.487
		Recessive	AG-GG	1.00		1.00	
			AA	1.19 (0.87–1.63)	0.289	1.19 (0.86–1.63)	0.290
		Additive	–	1.01 (0.85–1.21)	0.898	1.01 (0.85–1.21)	0.899
*MC4R*	rs6567160	Codominant	TT	1.00		1.00	
			CC	0.88 (0.53–1.47)	0.627	0.88 (0.53–1.47)	0.626
			CT	1.10 (0.84–1.44)	0.475	1.10 (0.84–1.44)	0.475
		Dominant	TT	1.00		1.00	
			CC-CT	1.06 (0.83–1.37)	0.634	1.06 (0.83–1.37)	0.635
		Recessive	CT-TT	1.00		1.00	
			CC	0.85 (0.51–1.41)	0.527	0.85 (0.51–1.41)	0.526
		Additive	–	1.01 (0.83–1.24)	0.899	1.01 (0.83–1.24)	0.900
*MC4R*	rs663129	Codominant	GG	1.00		1.00	
			AA	0.89 (0.53–1.48)	0.646	0.89 (0.53–1.48)	0.645
			AG	1.12 (0.86–1.47)	0.395	1.12 (0.86–1.47)	0.396
		Dominant	GG	1.00		1.00	
			AA-AG	1.08 (0.84–1.39)	0.545	1.08 (0.84–1.39)	0.546
		Recessive	AG-GG	1.00		1.00	
			AA	0.85 (0.51–1.41)	0.527	0.85 (0.51–1.41)	0.526
		Additive	–	1.02 (0.84–1.25)	0.818	1.02 (0.84–1.25)	0.820
*MC4R*	rs17782313	Codominant	TT	1.00		1.00	
			CC	0.89 (0.53–1.49)	0.664	0.89 (0.53–1.49)	0.663
			CT	1.14 (0.88–1.49)	0.324	1.14 (0.88–1.49)	0.324
		Dominant	TT	1.00		1.00	
			CC-CT	1.10 (0.85–1.42)	0.463	1.10 (0.85–1.41)	0.464
		Recessive	CT-TT	1.00		1.00	
			CC	0.85 (0.51–1.41)	0.527	0.85 (0.51–1.41)	0.526
		Additive	–	1.04 (0.85–1.27)	0.740	1.04 (0.85–1.27)	0.741
*MC4R*	rs12969709	Codominant	CC	1.00		1.00	
			AA	0.70 (0.40–1.22)	0.203	0.70 (0.40–1.21)	0.202
			AC	1.06 (0.81–1.38)	0.694	1.06 (0.81–1.38)	0.695
		Dominant	CC	1.00		1.00	
			AA-AC	0.99 (0.77–1.28)	0.969	0.99 (0.77–1.28)	0.967
		Recessive	AC-CC	1.00		1.00	
			AA	0.68 (0.40–1.18)	0.174	0.68 (0.40–1.18)	0.173
		Additive	–	0.94 (0.77–1.16)	0.578	0.94 (0.77–1.16)	0.577
*MC4R*	rs11663816	Codominant	TT	1.00		1.00	
			CC	0.88 (0.50–1.55)	0.659	0.88 (0.50–1.55)	0.657
			CT	0.98 (0.75–1.27)	0.854	0.98 (0.75–1.27)	0.852
		Dominant	TT	1.00		1.00	
			CC-CT	0.96 (0.75–1.24)	0.766	0.96 (0.75–1.24)	0.764
		Recessive	CT-TT	1.00		1.00	
			CC	0.89 (0.51–1.55)	0.678	0.89 (0.51–1.55)	0.676
		Additive	–	0.96 (0.78–1.18	0.688	0.96 (0.78–1.18)	0.686
*MC4R*	rs12970134	Codominant	GG	1.00		1.00	
			AA	0.83 (0.46–1.50)	0.543	0.83 (0.46–1.50)	0.542
			AG	0.97 (0.74–1.26)	0.801	0.97 (0.74–1.26)	0.800
		Dominant	GG	1.00		1.00	
			AA-AG	0.95 (0.73–1.23)	0.683	0.95 (0.73–1.23)	0.682
		Recessive	AG-GG	1.00		1.00	
			AA	0.84 (0.47–1.51)	0.564	0.84 (0.47–1.51)	0.564
		Additive	–	0.94 (0.76–1.17)	0.582	0.94 (0.76–1.17)	0.581

*SNP* single nucleotide polymorphism, *OR* odds ratio, *95% CI* 95% confidence interval

*p*^a^ values were calculated by logistic regression analysis with the comparison between diabetes patients and healthy controls

*p*^b^ values were calculated by logistic regression analysis with adjustment for age and gender

Bold values indicate statistical significance (*p* < 0.05)

Additionally, we investigated the correlation of *PDX1* and *MC4R* polymorphisms with T2DM risk in multiple inheritance models by logistic regression analyses (Table 2). The results revealed that the AG genotype of *PDX1*-rs9581943 decreased susceptibility to T2DM in the study subjects (OR = 0.76, 95% CI = 0.58–0.99, *p* = 0.045).

### Stratified analysis

Stratification analysis was carried out by age, sex, smoking, drinking and BMI. The results of stratification by age and sex are shown in Table 3. We found that *PDX1*-rs9581943 significantly decreased the risk of T2DM among patients aged ≤ 60 years in the codominant (OR = 0.66, 95% CI = 0.45–0.98, *p* = 0.039) and dominant models (OR = 0.69, 95% CI = 0.48–1.00, *p* = 0.049). Rs6567160, rs663129, rs17782313, rs12969709 and rs11663816 in *MC4R* reduced the susceptibility to T2DM among individuals aged ≤ 60 years under the codominant (rs6567160: OR = 0.33, 95% CI = 0.13–0.81, *p* = 0.015; rs663129: OR = 0.33, 95% CI = 0.13–0.82, *p* = 0.017; rs17782313: OR = 0.34, 95% CI = 0.14–0.83, *p* = 0.018; rs12969709: OR = 0.27, 95% CI = 0.10–0.75, *p* = 0.012; rs11663816: OR = 0.31, 95% CI = 0.11–0.88, *p* = 0.027) and recessive (rs6567160: OR = 0.33, 95% CI = 0.14–0.81, *p* = 0.016; rs663129: OR = 0.33, 95% CI = 0.14–0.81, *p* = 0.016; rs17782313: OR = 0.33, 95% CI = 0.14–0.81, *p* = 0.016; rs12969709: OR = 0.27, 95% CI = 0.10–0.75, *p* = 0.012; and rs11663816: OR = 0.32, 95% CI = 0.11–0.91, *p* = 0.032) models. After stratifying by sex, rs9581943 (OR = 0.73, 95% CI = 0.5–1.00, *p* = 0.049) and rs7981781 (OR = 0.70, 95% CI = 0.56–0.97, *p* = 0.033) were found to be associated with a decreased risk of T2DM in males under the codominant model.

**Table 3:** Relationships of *PDX1 and MC4R* polymorphisms with T2DM risk stratified by age and sex

Gene SIP	Model	Genotype	> 60		≤ 60		Male		Female	
			OR (95% CI)	*p*	OR (95% CI)	*p*	OR (95% CI)	*p*	OR (95% CI)	*p*
*PDX1* rs9581943	Allele	G	1.00		1.00		1.00		1.00	
		A	0.99 (0.76–1.28)	0.919	0.84 (0.65–1.09)	0.180	0.94 (0.75–1.16)	0.544	0.87 (0.62–1.22)	0.424
	Codominant	GG	1.00		1.00		1.00		1.00	
		AA	1.05 (0.60–1.84)	0.852	0.79 (0.45–1.38)	0.409	1.05 (0.67–1.66)	0.829	0.77 (0.37–1.60)	0.481
		AG	0.86 (0.58–1.27)	0.439	0.66 (0.45–0.98)	**0.039**	0.73 (0.5–1.00)	**0.049**	0.85 (0.51–1.40)	0.516
	Dominant	GG	1.00		1.00		1.00		1.00	
		AA-AG	0.90 (0.63–1.30)	0.580	0.69 (0.48–1.00)	**0.049**	0.80 (0.59–1.07)	0.130	0.83 (0.51–1.34)	0.439
	Recessive	AG-GG	1.00		1.00		1.00		1.00	
		AA	1.14 (0.67–1.92)	0.634	0.99 (0.59–1.67)	0.982	1.24 (0.81–1.89)	0.330	0.84 (0.43–1.66)	0.620
	Additive	–	0.98 (0.76–1.27)	0.873	0.83 (0.63–1.08)	0.156	0.94 (0.76–1.16)	0.554	0.87 (0.62–1.22)	0.421
*PDX1* rs7981781	Allele	G	1.00		1.00		1.00		1.00	
		A	0.96 (0.75–1.23)	0.753	1.08 (0.84–1.40)	0.542	0.94 (0.76–1.16)	0.558	1.22 (0.88–1.70)	0.241
	Codominant	GG	1.00		1.00		1.00		1.00	
		AA	1.00 (0.60–1.67)	0.999	1.23 (0.72–2.08)	0.449	0.96 (0.64–1.46)	0.856	1.46 (0.72–2.96)	0.300
		AG	0.89 (0.59–1.35)	0.584	0.91 (0.61–1.35)	0.628	0.70 (0.50–0.97)	**0.033**	1.41 (0.83–2.39)	0.203
	Dominant	GG	1.00		1.00		1.00		1.00	
		AA-AG	0.92 (0.62–1.36)	0.683	0.98 (0.67–1.43)	0.925	0.77 (0.56–1.05)	0.096	1.42 (0.86–2.35)	0.172
	Recessive	AG-GG	1.00		1.00		1.00		1.00	
		AA	1.07 (0.69–1.68)	0.757	1.30 (0.81–2.09)	0.281	1.19 (0.83–1.72)	0.352	1.18 (0.63–2.20)	0.615
	Additive	–	0.99 (0.77–1.27)	0.928	1.07 (0.83–1.38)	0.603	0.94 (0.77–1.16)	0.567	1.24 (0.88–1.75)	0.225
*MC4R* rs6567160	Allele	T	1.00		1.00		1.00		1.00	
		C	1.32 (0.99–1.75)	0.060	0.77 (0.57–1.04)	0.091	0.96 (0.7–1.23)	0.756	1.16 (0.79–1.71)	0.460
	Codominant	TT	1.00		1.00		1.00		1.00	
		CC	1.81 (0.91–3.58)	0.091	0.33 (0.13–0.81)	**0.015**	0.70 (0.37–1.32)	0.271	1.41 (0.57–3.54)	0.459
		CT	1.22 (0.82–1.80)	0.322	0.96 (0.65–1.40)	0.815	1.11 (0.81–1.52)	0.522	1.08 (0.65–1.81)	0.758
	Dominant	TT	1.00		1.00		1.00		1.00	
		CC-CT	1.31 (0.91–1.89)	0.144	0.84 (0.58–1.21)	0.340	1.04 (0.77–1.39)	0.820	1.14 (0.70–1.84)	0.596
	Recessive	CT-TT	1.00		1.00		1.00		1.00	
		CC	1.68 (0.86–3.28)	0.129	0.33 (0.14–0.81)	**0.016**	0.68 (0.36–1.26)	0.215	1.37 (0.56–3.37)	0.489
	Additive	–	1.29 (0.97–1.71)	0.077	0.77 (0.57–1.04)	0.085	0.96 (0.76–1.23)	0.760	1.14 (0.79–1.66)	0.484
*MC4R* rs663129	Allele	G	1.00		1.00		1.00		1.00	
		A	1.32 (0.99–1.75)	0.060	0.79 (0.59–1.07)	0.125	0.98 (0.77–1.25)	0.852	1.16 (0.79–1.71)	0.460
	Codominant	GG	1.00		1.00		1.00		1.00	
		AA	1.81 (0.91–3.58)	0.091	0.33 (0.13–0.82)	**0.017**	0.71 (0.38–1.33)	0.284	1.41 (0.57–3.54)	0.459
		AG	1.22 (0.82–1.80)	0.322	0.99 (0.68–1.45)	0.966	1.14 (0.83–1.55)	0.424	1.08 (0.65–1.81)	0.758
	Dominant	GG	1.00		1.00		1.00		1.00	
		AA-AG	1.31 (0.91–1.89)	0.144	0.87 (0.60–1.25)	0.441	1.06 (0.79–1.43)	0.704	1.14 (0.70–1.84)	0.596
	Recessive	AG-GG	1.00		1.00		1.00		1.00	
		AA	1.68 (0.86–3.28)	0.129	0.33 (0.14–0.81)	**0.016**	0.68 (0.36–1.26)	0.215	1.37 (0.56–3.37)	0.489
	Additive	–	1.29 (0.97–1.71)	0.077	0.78 (0.58–1.06)	0.116	0.98 (0.77–1.24)	0.854	1.14 (0.79–1.66)	0.484
*MC4R* rs17782313	Allele	T	1.00		1.00		1.00		1.00	
		C	1.32 (0.99–1.75)	0.060	0.81 (0.60–1.09)	0.167	0.98 (0.77–1.26)	0.901	1.18 (0.80–1.74)	0.403
	Codominant	TT	1.00		1.00		1.00		1.00	
		CC	1.81 (0.91–3.58)	0.091	0.34 (0.14–0.83)	**0.018**	0.71 (0.38–1.34)	0.291	1.43 (0.57–3.58)	0.443
		CT	1.22 (0.82–1.80)	0.322	1.03 (0.71–1.51)	0.867	1.15 (0.84–1.58)	0.378	1.12 (0.67–1.87)	0.660
	Dominant	TT	1.00		1.00		1.00		1.00	
		CC-CT	1.31 (0.91–1.89)	0.144	0.90 (0.62–1.30)	0.569	1.07 (0.80–1.44)	0.648	1.17 (0.73–1.90)	0.515
	Recessive	CT-TT	1.00		1.00		1.00		1.00	
		CC	1.68 (0.86–3.28)	0.129	0.33 (0.14–0.81)	**0.016**	0.68 (0.36–1.26)	0.215	1.37 (0.56–3.37)	0.489
	Additive	–	1.29 (0.97–1.71)	0.077	0.80 (0.59–1.09)	0.159	0.99 (0.77–1.25)	0.903	1.16 (0.80–1.69)	0.426
*MC4R* rs12969709	Allele	C	1.00		1.00		1.00		1.00	
		A	1.13 (0.85–1.51)	0.406	0.78 (0.57–1.06)	0.111	0.91 (0.71–1.16)	0.449	1.03 (0.69–1.53)	0.884
	Codominant	CC	1.00		1.00		1.00		1.00	
		AA	1.37 (0.67–2.80)	0.396	0.27 (0.10–0.75)	**0.012**	0.58 (0.29–1.15)	0.117	1.02 (0.39–2.70)	0.965
		AC	1.12 (0.76–1.65)	0.583	0.98 (0.67–1.43)	0.900	1.06 (0.77–1.45)	0.726	1.05 (0.63–1.75)	0.867
	Dominant	CC	1.00		1.00		1.00		1.00	
		AA-AC	1.16 (0.80–1.67)	0.442	0.85 (0.59–1.23)	0.397	0.98 (0.72–1.32)	0.879	1.04 (0.64–1.69)	0.871
	Recessive	AC-CC	1.00		1.00		1.00		1.00	
		AA	1.31 (0.65–2.66)	0.449	0.27 (0.10–0.75)	**0.012**	0.57 (0.29–1.11)	0.099	1.01 (0.39–2.62)	0.989
	Additive	–	1.14 (0.86–1.53)	0.361	0.77 (0.56–1.05)	0.098	0.91 (0.71–1.16)	0.451	1.03 (0.70–1.51)	0.893
*MC4R* rs11663816	Allele	T	1.00		1.00		1.00		1.00	
		C	1.21 (0.90–1.61)	0.203	0.75 (0.55–1.02)	0.067	0.92 (0.72–1.17)	0.488	1.07 (0.72–1.60)	0.727
	Codominant	TT	1.00		1.00		1.00		1.00	
		CC	1.78 (0.85–3.73)	0.127	0.31 (0.11–0.88)	**0.027**	0.70 (0.35–1.39)	0.304	1.44 (0.53–3.96)	0.476
		CT	1.07 (0.73–1.58)	0.726	0.88 (0.60–1.28)	0.504	0.98 (0.72–1.34)	0.923	0.94 (0.57–1.57)	0.823
	Dominant	TT	1.00		1.00		1.00		1.00	
		CC-CT	1.17 (0.81–1.68)	0.412	0.80 (0.55–1.15)	0.223	0.94 (0.70–1.27)	0.703	1.01 (0.62–1.64)	0.968
	Recessive	CT-TT	1.00		1.00		1.00		1.00	
		CC	1.74 (0.84–3.59)	0.137	0.32 (0.11–0.91)	**0.032**	0.70 (0.35–1.38)	0.305	1.47 (0.54–3.98)	0.447
	Additive	–	1.21 (0.90–1.61)	0.210	0.75 (0.55–1.03)	0.072	0.92 (0.71–1.17)	0.486	1.07 (0.73–1.57)	0.739

*SNP* single nucleotide polymorphism, *OR* odds ratio, *95% CI* 95% confidence interval

*p* values were calculated by logistic regression analysis with adjustment for age and gender

Bold values indicate statistical significance (*p* < 0.05)

In addition, as shown in Table 4, *PDX1*-rs7981781 reduced the susceptibility to T2DM among smokers under the codominant (OR = 0.50, 95% CI = 0.29–0.89, *p* = 0.018) and dominant (OR = 0.55, 95% CI = 0.32–0.95, *p* = 0.030) models. However, *MC4R*-rs6567160 could increase the occurrence of T2DM among nonsmokers under the codominant (OR = 1.60, 95% CI = 1.04–2.45, *p* = 0.032) and dominant (OR = 1.56, 95% CI = 1.04–2.34, *p* = 0.031) models. *MC4R*-rs663129 induced a significantly higher susceptibility to T2DM among individuals who were nonsmokers in the codominant (OR = 1.64, 95% CI = 1.07–2.52, *p* = 0.023), dominant (OR = 1.60, 95% CI = 1.07–2.40, *p* = 0.023) and additive (OR = 1.40, 95% CI = 1.00–1.95, *p* = 0.049) models. Moreover, rs17782313 in *MC4R* was related to a higher risk of T2DM among nonsmokers under the allelic (OR = 1.43, 95% CI = 1.00–1.95, *p* = 0.036), codominant (OR = 1.72, 95% CI = 1.12–2.64, *p* = 0.014), dominant (OR = 1.66, 95% CI = 1.11–2.50, *p* = 0.014) and additive (OR = 1.44, 95% CI = 1.03–2.01, *p* = 0.034) models.

**Table 4:** The associations between *PDX1* and *MC4R* polymorphisms and the risk of T2DM stratified by smoking, drinking status

Gene SIP	Model	Genotype	Smoking		Non-smoking		Drinking		Non-drinking	
			OR (95% CI)	*p*	OR (95% CI)	*p*	OR (95% CI)	*p*	OR (95% CI)	*p*
*PDX1* rs11619319	Allele	A	1.00		1.00		1.00		1.00	
		G	0.82 (0.58–1.15)	0.246	0.92 (0.70–1.21)	0.535	0.80 (0.55–1.18)	0.263	0.93 (0.71–1.22)	0.608
	Codominant	AA	1.00		1.00		1.00		1.00	
		GG	0.75 (0.37–1.50)	0.410	0.84 (0.48–1.49)	0.558	0.67 (0.30–1.47)	0.313	0.89 (0.50–1.56)	0.676
		GA	0.61 (0.34–1.09)	0.098	0.77 (0.48–1.23)	0.274	0.51 (0.27–0.97)	**0.039**	0.81 (0.51–1.29)	0.381
	Dominant	AA	1.00		1.00		1.00		1.00	
		GG-GA	0.65 (0.38–1.13)	0.125	0.79 (0.51–1.23)	0.299	0.55 (0.30–1.01)	0.054	0.83 (0.54–1.29)	0.418
	Recessive	GA-AA	1.00		1.00		1.00		1.00	
		GG	1.02 (0.57–1.83)	0.943	1.00 (0.62–1.62)	0.998	1.01 (0.63–1.63)	0.957	1.01 (0.63–1.63)	0.957
	Additive	–	0.85 (0.61–1.19)	0.346	0.91 (0.69–1.20)	0.501	0.93 (0.71–1.23)	0.623	0.93 (0.71–1.23)	0.623
*PDX1* rs2293941	Allele	G	1.00		1.00		1.00		1.00	
		A	0.83 (0.59–1.16)	0.274	0.91 (0.69–1.20)	0.515	0.80 (0.55–1.18)	0.264	0.94 (0.71–1.23)	0.647
	Codominant	GG	1.00		1.00		1.00			
		AA	0.77 (0.38–1.54)	0.454	0.85 (0.48–1.50)	0.568	0.68 (0.31–1.48)	0.326	0.90 (0.51–1.57)	0.703
		AG	0.64 (0.36–1.13)	0.124	0.80 (0.50–1.26)	0.331	0.51 (0.27–0.97)	**0.040**	0.85 (0.54–1.34)	0.477
	Dominant	GG	1.00		1.00		1.00		1.00	
		AA-AG	0.67 (0.39–1.16)	0.156	0.81 (0.52–1.25)	0.345	0.56 (0.30–1.02)	0.056	0.86 (0.56–1.33)	0.499
	Recessive	AG-GG	1.00		1.00		1.00		1.00	
		AA	1.02 (0.57–1.83)	0.943	0.98 (0.61–1.59)	0.939	1.03 (0.53–2.00)	0.935	1.00 (0.62–1.61)	0.986
	Additive	–	0.86 (0.62–1.21)	0.384	0.91 (0.69–1.20)	0.511	0.79 (0.54–1.16)	0.231	0.94 (0.71–1.24)	0.654
*PDX1* rs7981781	Allele	G	1.00		1.00		1.00		1.00	
		A	0.76 (0.54–1.07)	0.117	0.95 (0.72–1.25)	0.726	0.73 (0.50–1.08)	0.111	0.96 (0.73–1.27)	0.786
	Codominant	GG	1.00		1.00		1.00		1.00	
		AA	0.68 (0.34–1.36)	0.277	0.92 (0.53–1.61)	0.775	0.57 (0.26–1.26)	0.167	0.94 (0.54–1.64)	0.834
		AG	0.50 (0.29–0.89)	**0.018**	0.89 (0.57–1.41)	0.628	0.47 (0.25–0.88)	**0.019**	0.92 (0.59–1.44)	0.716
	Dominant	GG	1.00		1.00		1.00		1.00	
		AA-AG	0.55 (0.32–0.95)	**0.030**	0.90 (0.59–1.38)	0.636	0.49 (0.27–0.90)	**0.022**	0.93 (0.61–1.41)	0.724
	Recessive	AG-GG	1.00		1.00		1.00		1.00	
		AA	1.03 (0.57–1.86)	0.919	0.99 (0.61–1.60)	0.962	0.92 (0.47–1.81)	0.811	0.99 (0.61–1.60)	0.972
	Additive	–	0.80 (0.57–1.11)	0.175	0.95 (0.72–1.26)	0.737	0.72 (0.49–1.07)	0.100	0.97 (0.74–1.27)	0.803
*MC4R* rs6567160	Allele	T	1.00		1.00		1.00		1.00	
		C	0.92 (0.61–1.37)	0.665	1.36 (0.98–1.90)	0.068	0.90 (0.56–1.46)	0.682	1.14 (0.83–1.58)	0.416
	Codominant	TT	1.00		1.00		1.00		1.00	
		CC	0.99 (0.33–2.93)	0.980	1.37 (0.58–3.20)	0.472	1.13 (0.29–4.41)	0.857	0.99 (0.45–2.18)	0.988
		CT	0.86 (0.52–1.44)	0.569	1.60 (1.04–2.45)	**0.032**	0.80 (0.44–1.45)	0.460	1.34 (0.87–2.04)	0.180
	Dominant	TT	1.00		1.00		1.00		1.00	
		CC-CT	0.88 (0.54–1.43)	0.601	1.56 (1.04–2.34)	**0.031**	0.83 (0.47–1.47)	0.532	1.27 (0.85–1.90)	0.237
	Recessive	CT-TT	1.00		1.00		1.00		1.00	
		CC	1.04 (0.36–3.04)	0.942	1.16 (0.50–2.69)	0.725	1.22 (0.32–4.67)	0.776	0.89 (0.41–1.92)	0.772
	Additive	–	0.92 (0.62–1.37)	0.688	1.37 (0.99–1.92)	0.061	0.90 (0.56–1.45)	0.673	1.15 (0.83–1.58)	0.407
*MC4R* rs663129	Allele	G	1.00		1.00		1.00		1.00	
		A	0.93 (0.62–1.38)	0.713	1.39 (0.99–1.94)	0.053	0.96 (0.59–1.55)	0.869	1.14 (0.83–1.58)	0.416
	Codominant	GG	1.00		1.00		1.00		1.00	
		AA	0.99 (0.33–2.95)	0.990	1.38 (0.59–3.23)	0.459	1.17 (0.30–4.54)	0.825	0.99 (0.45–2.18)	0.988
		AG	0.88 (0.53–1.47)	0.624	1.64 (1.07–2.52)	**0.023**	0.88 (0.48–1.58)	0.660	1.34 (0.87–2.04)	0.180
	Dominant	GG	1.00		1.00		1.00		1.00	
		AA-AG	0.89 (0.55–1.46)	0.655	1.60 (1.07–2.40)	**0.023**	0.91 (0.51–1.60)	0.735	1.27 (0.85–1.90)	0.237
	Recessive	AG-GG	1.00		1.00		1.00		1.00	
		AA	1.04 (0.36–3.04)	0.942	1.16 (0.50–2.69)	0.725	1.22 (0.32–4.67)	0.776	0.89 (0.41–1.92)	0.772
	Additive	–	0.93 (0.63–1.39)	0.734	1.40 (1.00–1.95)	**0.049**	0.96 (0.60–1.54)	0.855	1.15 (0.83–1.58)	0.407
*MC4R* rs17782313	Allele	T	1.00		1.00		1.00		1.00	
		C	0.93 (0.62–1.38)	0.713	1.43 (1.02–2.00)	**0.036**	0.96 (0.59–1.55)	0.869	1.18 (0.85–1.63)	0.329
	Codominant	TT	1.00		1.00		1.00		1.00	
		CC	0.99 (0.33–2.95)	0.990	1.40 (0.60–3.28)	0.439	1.17 (0.30–4.54)	0.825	1.01 (0.46–2.21)	0.977
		CT	0.88 (0.53–1.47)	0.624	1.72 (1.12–2.64)	**0.014**	0.88 (0.48–1.58)	0.660	1.40 (0.92–2.15)	0.118
	Dominant	TT	1.00		1.00		1.00		1.00	
		CC-CT	0.89 (0.55–1.46)	0.655	1.66 (1.11–2.50)	**0.014**	0.91 (0.51–1.60)	0.735	1.33 (0.89–1.99)	0.163
	Recessive	CT-TT	1.00		1.00		1.00		1.00	
		CC	1.04 (0.36–3.04)	0.942	1.16 (0.50–2.69)	0.725	1.22 (0.32–4.67)	0.776	0.89 (0.41–1.92)	0.772
	Additive	–	0.93 (0.63–1.39)	0.734	1.44 (1.03–2.01)	**0.034**	0.96 (0.60–1.54)	0.855	1.18 (0.85–1.63)	0.317

*SNP* single nucleotide polymorphism, *OR* odds ratio, *95% CI* 95% confidence interval

*p* values were calculated by logistic regression analysis with adjustment for age and gender

Bold values indicate statistical significance (*p* < 0.05)

Additionally, *PDX1*-rs11619319 (OR = 0.51, 95% CI = 0.27–20.97, *p* = 0.039) and rs2293941 (OR = 0.51, 95% CI = 0.27–0.97, *p* = 0.040) were predominantly related to a reduced risk of T2DM among drinkers under the codominant model. Rs7981781 was correlated with a lower risk of T2DM among drinkers under the codominant (OR = 0.47, 95% CI = 0.25–0.88, *p* = 0.019) and dominant (OR = 0.49, 95% CI = 0.27–0.90, *p* = 0.022) models.

When stratified by BMI (Table 5), *PDX1*-rs7981781 was correlated with a lower risk of T2DM among subjects with BMI > 24 kg/m^2^ under the codominant model (OR = 0.64, 95% CI = 0.41–1.00, *p* = 0.049).

**Table 5:** The association between *PDX1* polymorphisms and the risk of T2DM stratified by BMI

Gene SIP	Model	Genotype	≤ 24		> 24	
			OR (95% CI)	*p*	OR (95% CI)	*p*
*PDX1* rs7981781	Allele	G	1.00		1.00	
		A	1.07 (0.78–1.46)	0.675	0.94 (0.72–1.24)	0.682
	Codominant	GG	1.00		1.00	
		AA	1.10 (0.59–2.06)	0.766	0.97 (0.54–1.72)	0.910
		AG	0.98 (0.58–1.64)	0.928	0.64 (0.41–1.00)	**0.049**
	Dominant	GG	1.00		1.00	
		AA-AG	1.01 (0.62–1.65)	0.956	0.72 (0.47–1.09)	0.117
	Recessive	AG-GG	1.00		1.00	
		AA	1.12 (0.65–1.92)	0.690	1.26 (0.75–2.10)	0.383
	Additive	–	1.04 (0.76–1.43)	0.790	0.92 (0.70–1.22)	0.559

*SNP* single nucleotide polymorphism, *OR* odds ratio, *95% CI* 95% confidence interval

*p* values were calculated by logistic regression analysis with adjustment for age and gender

Bold values indicate statistical significance (*p* < 0.05)

### Haplotype analysis

**Fig. 1:**
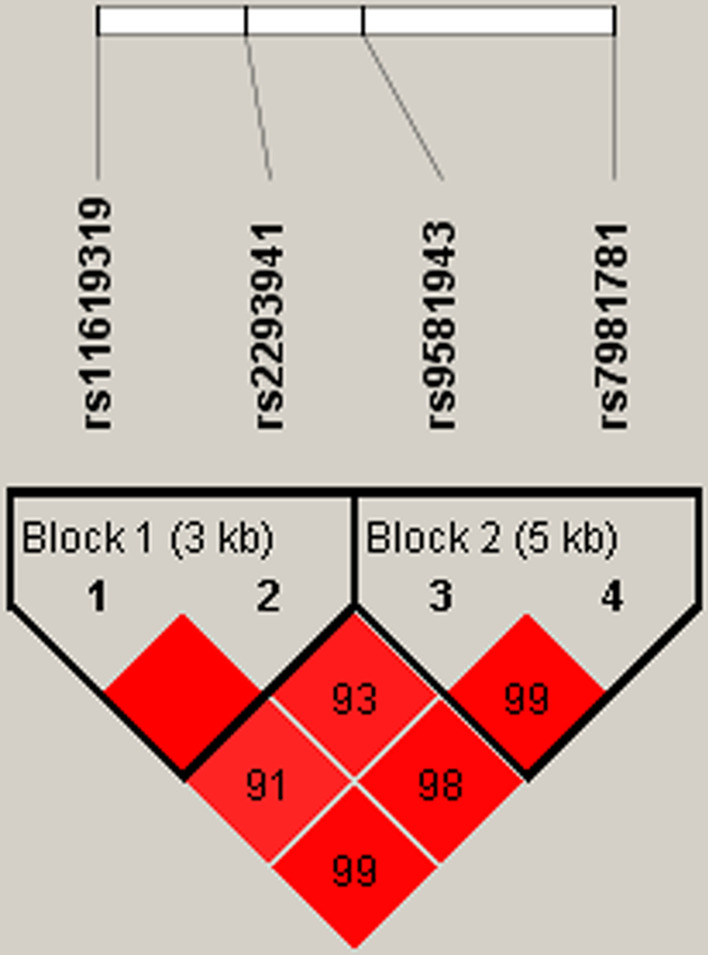
Haplotype block map for SNPs in *PDX1*. Block 1 includes rs11619319 and rs2293941. Block 2 includes rs9581943 and rs7981781. The numbers inside the diamonds indicate the D’ for pairwise analyses

**Fig. 2:**
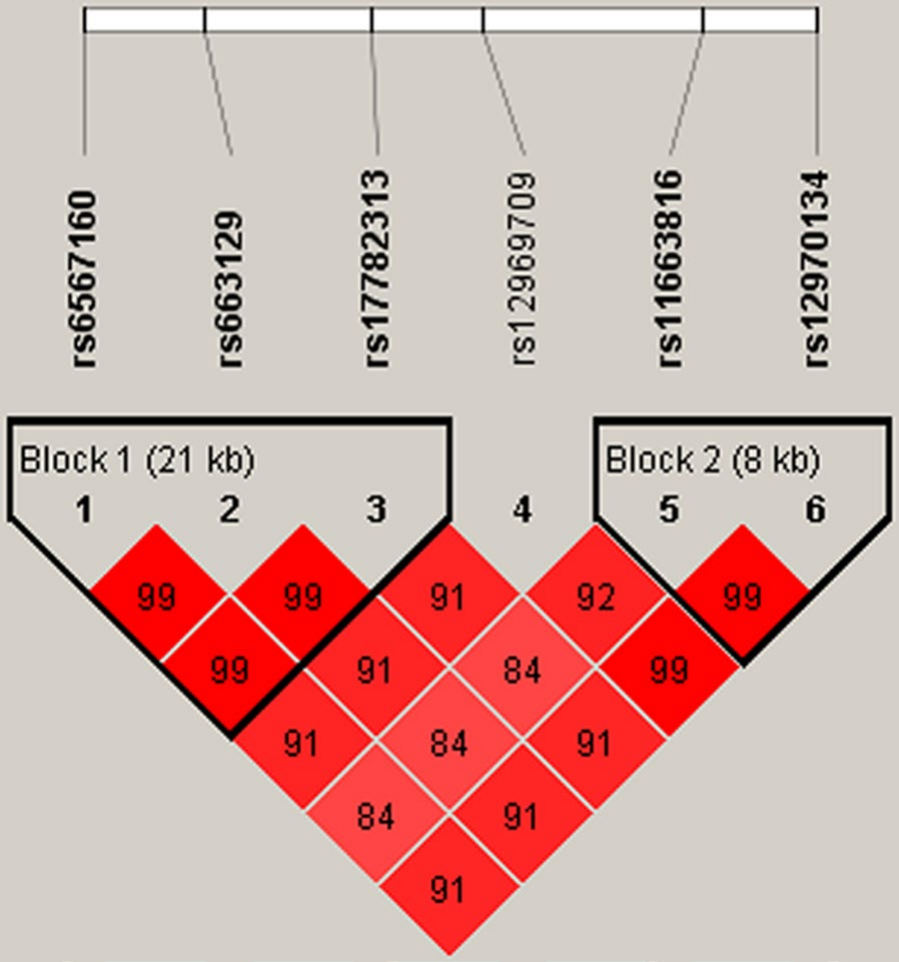
Haplotype block map for SNPs in *MC4R*.Block 1 includes rs6567160, rs663129 and rs17782313. Block 2 includes rs11663816 and rs12970134. The numbers inside the diamonds indicate the D’ for pairwise analyses

We next conducted linkage disequilibrium (LD) analysis for the polymorphisms in *MC4R1* and *PDX1*. Our results indicated two blocks (block1: rs11619319 and rs2293941; block2: rs9581943 and rs7981781) in *PDX1* (Fig. 1) and two blocks (block1: rs6567160, rs663129, and rs17782313; block2: rs11663816 and rs12970134) in *MC4R* (Fig. 2)*.* Besides, Table 6 shows that there was no association between haplotype frequency and T2DM risk (*p* > 0.05).

**Table 6:** Haplotype analysis of *PDX1* and *MC4R* SNPs with T2DM risk

Gene	SNP	Haplotype	Frequency in cases	Frequency in controls	With adjustment		Without adjustment	
					OR (95%CI)	*p*	OR (95%CI)	*p*
*PDX1*	rs11619319|rs2293941	GA	0.446	0.440	1.02 (0.86–1.22)	0.791	1.02 (0.86–1.22)	0.792
*PDX1*	rs11619319|rs2293941	AG	0.451	0.444	1.03 (0.86–1.23)	0.755	1.03 (0.86–1.23)	0.756
*PDX1*	rs9581943|rs7981781	GA	0.432	0.431	1.00 (0.84–1.20)	0.969	1.00 (0.84–1.20)	0.970
*PDX1*	rs9581943|rs7981781	AG	0.350	0.372	0.91 (0.72–1.09)	0.307	0.91 (0.76–1.09)	0.308
*PDX1*	rs9581943|rs7981781	GG	0.216	0.197	1.13 (0.91–1.40)	0.283	1.13 (0.91–1.40)	0.284
*MC4R*	rs6567160|rs663129|rs17782313	CAC	0.237	0.233	1.02 (0.84–1.25)	0.819	1.02 (0.84–1.25)	0.820
*MC4R*	rs6567160|rs663129|rs17782313	TGT	0.239	0.235	1.02 (0.84–1.25)	0.818	1.02 (0.84–1.25)	0.819
*MC4R*	rs11663816|rs12970134	CA	0.204	0.217	0.93 (0.75–1.15)	0.510	0.93 (0.75–1.15)	0.509
*MC4R*	rs11663816|rs12970134	CG	0.015	0.011	1.38 (0.63–3.04)	0.423	1.38 (0.63–3.04)	0.423
*MC4R*	rs11663816|rs12970134	TG	0.221	0.228	0.97 (0.78–1.19)	0.746	0.97 (0.78–1.19)	0.744

*SNP* single nucleotide polymorphism, *OR* odd ratios, *CI* confidence interval

### The relative mRNA expression of *PDX1* and *MC4R*

**Fig. 3:**
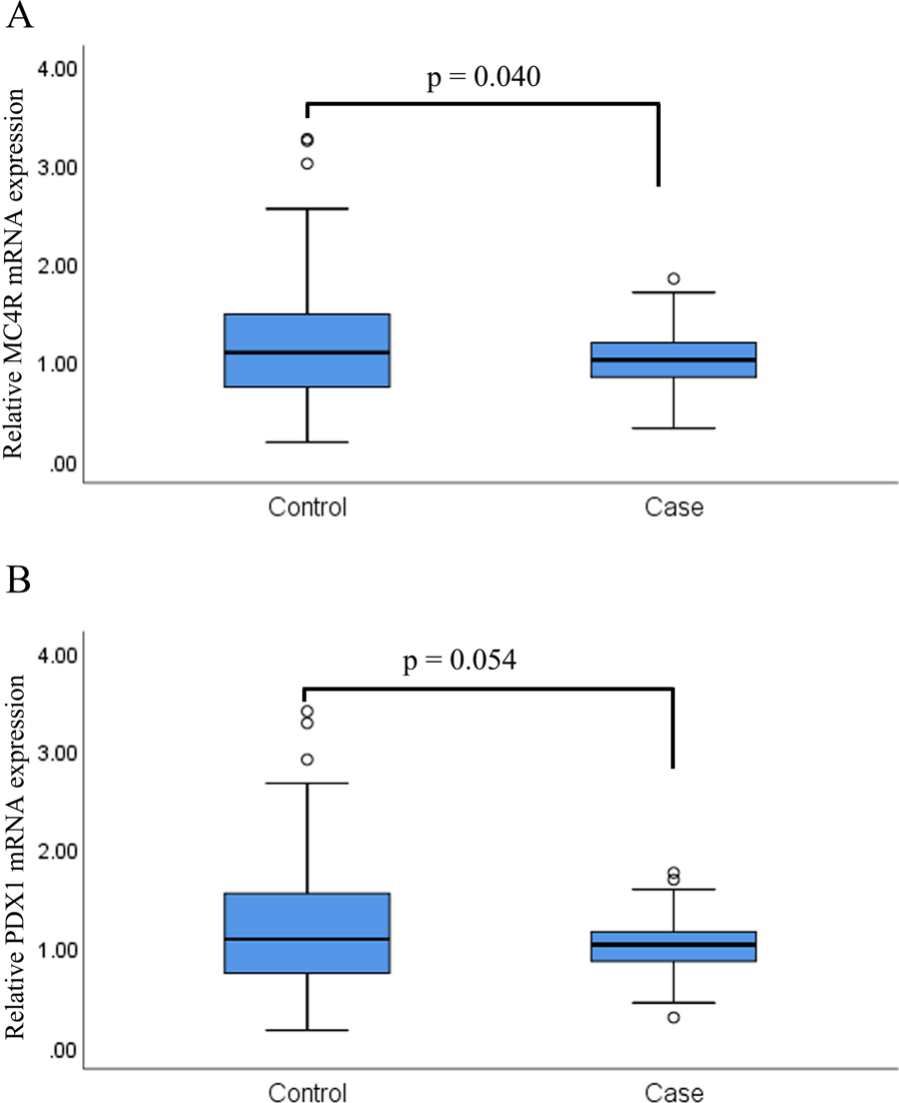
The relative mRNA expression of the *MC4R* and *PDX1* genes in T2DM patients and controls. T2DM, type 2 diabetes mellitus

The *MC4R* mRNA expression levels in T2DM case subjects decreased compared with those in their nondiabetic counterparts (*p* = 0.040, Fig. 3a). In addition, although no significant differences were observed in the expression levels of *PDX1* mRNA between the two groups, we did observe a decreased pattern of *PDX1* expression in individual samples between the cases and controls (*p* = 0.054, Fig. 3b).

### The association of relative mRNA expression and *PDX1* and *MC4R* polymorphisms

**Fig. 4:**
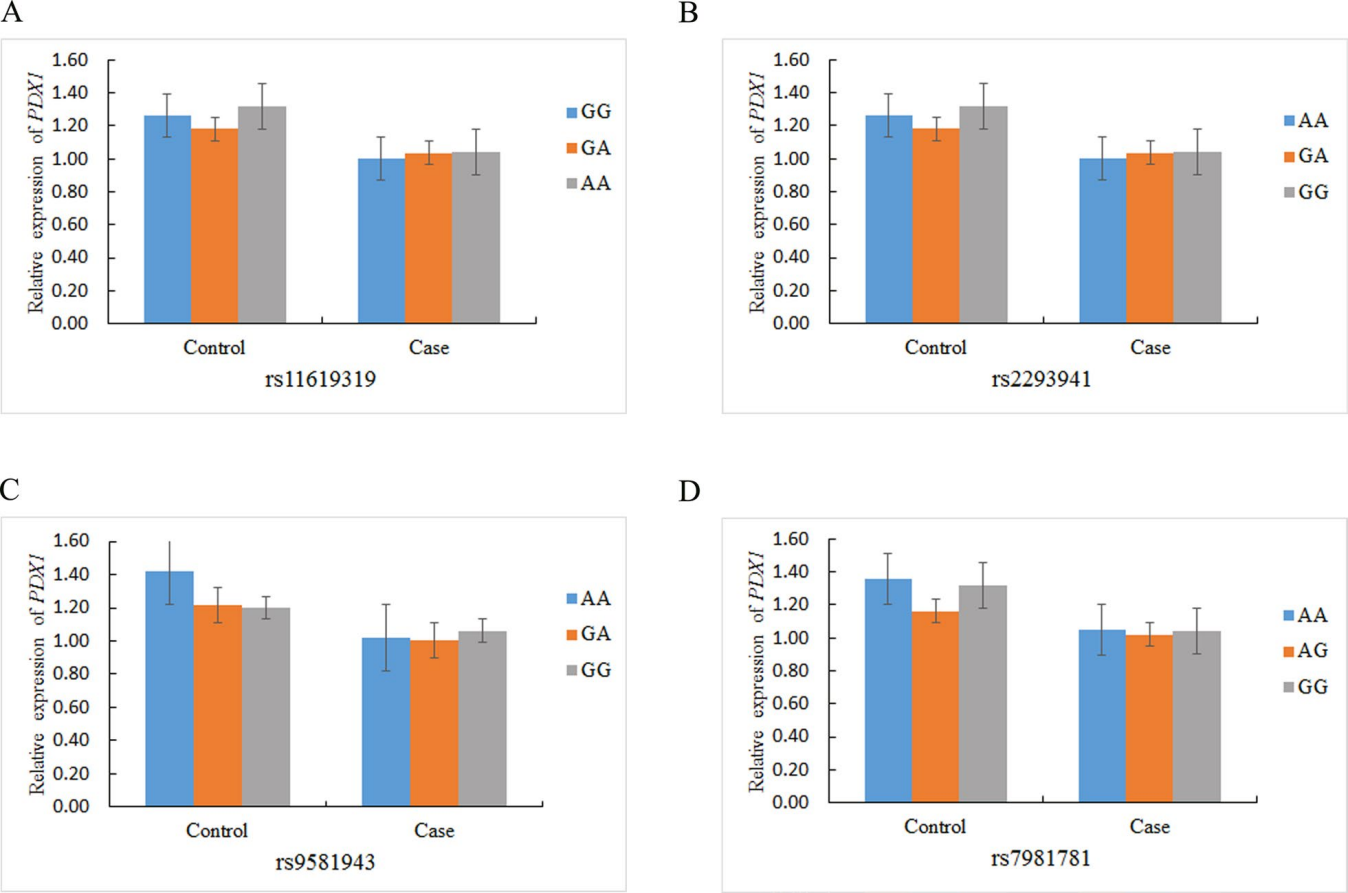
The association of relative *PDX1* mRNA expression and genetic polymorphisms in T2DM patients and healthy controls

**Fig. 5:**
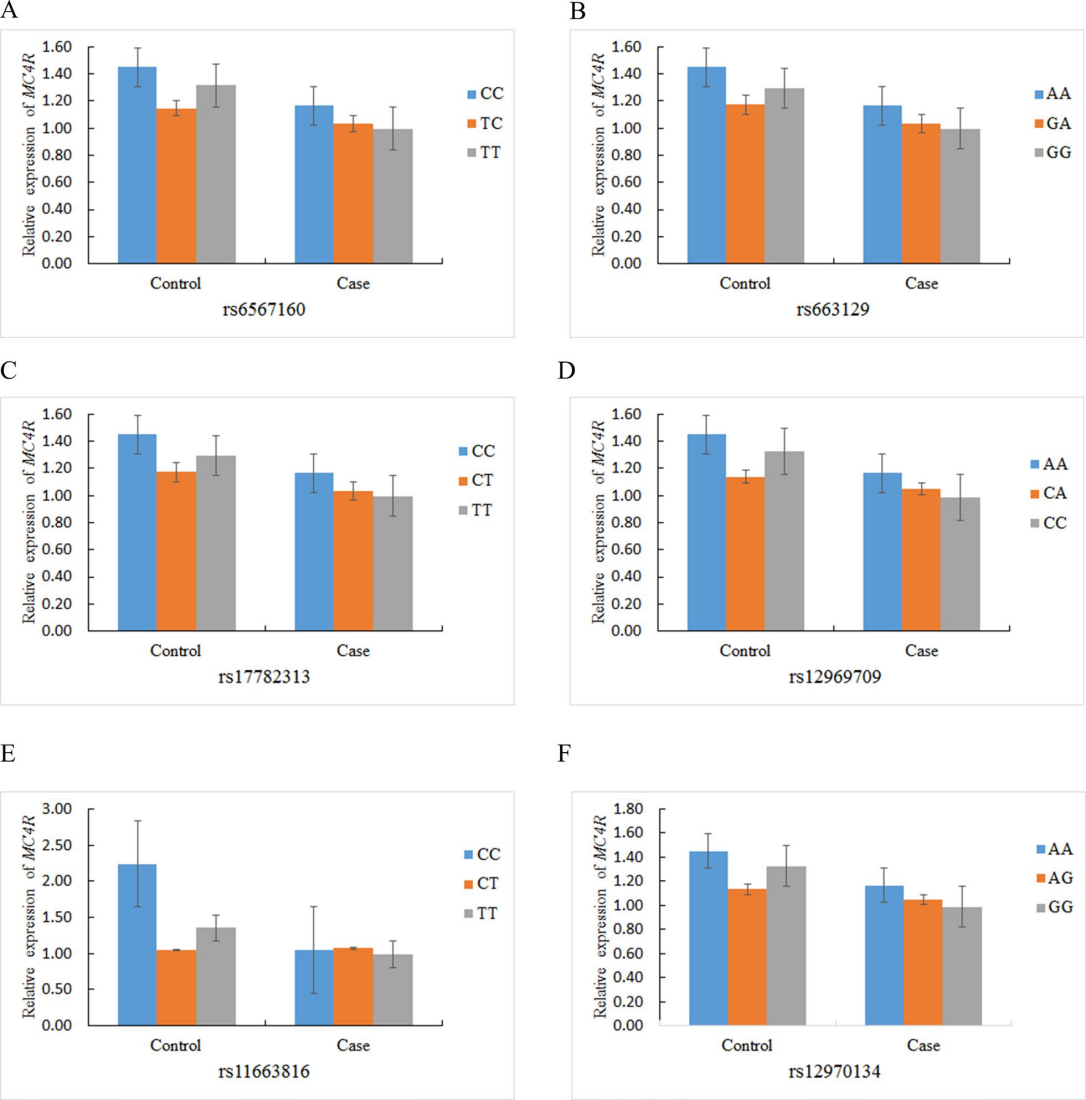
The association of relative *MC4R* mRNA expression and genetic polymorphisms in T2DM patients and healthy controls

The *PDX1* and *MC4R* polymorphisms were not associated with the relative *PDX1* and *MC4R* mRNA expression in the T2DM patients and controls (Figs. 4, 5).

## Discussion

This research focused on the association of *PDX1* and *MC4R* polymorphisms with susceptibility to T2DM in Chinese Han people. We found that *PDX1*-rs9581943 was correlated with a decreased risk of T2DM among the study subjects. In addition, the effects of *PDX1* and *MC4R* polymorphisms on T2DM susceptibility were dependent on age, sex, smoking status, drinking status and BMI. These findings suggest that genetic polymorphisms in *PDX1* and *MC4R* may play a crucial role in the development of T2DM.

In humans, the *PDX1* gene is located on chromosome 13q12.1. It is a key transcription factor involved in pancreatic development, islet hormone and insulin expression. Data from several studies suggested that deletion and mutation in *PDX1* caused overt diabetes and maturity-onset diabetes of the young [21, 22]. Additionally, Steinthorsdottir et al. found that rare frameshift variants in *PDX1* were associated with a higher risk of T2DM in Icelanders [6]. Recently, a homozygous mutation in *PDX1* was detected in a 65-day-old Iranian patient with neonatal diabetes [23]. However, there are few studies on rs11619319, rs2293941, rs9581943, and rs7981781. In the present study, we found that only rs9581943 decreased the incidence of T2DM among the study subjects. Moreover, we found that the relative mRNA expression of the *PDX1* gene was lower in T2DM patients than in controls, but the difference was insignificant. Interestingly, stratified analysis results revealed that rs9581943, rs11619319, rs2293941, and rs7981781were associated with susceptibility to T2DM in different subgroups. Manning et al. [24] illustrated that rs2293941 was associated with fasting glucose levels in individuals of European ancestry. However, this correlation was not observed among participants in the Chinese Han population in the present study (not shown). The inconsistencies in these reports may result from subjects of different ethnicities and different environments. Taken together, these results demonstrated that the *PDX1* polymorphism is important in the development and risk assessment of T2DM.

*MC4R* is a G-protein-coupled receptor that is highly expressed in the hypothalamus, where it regulates appetite, energy expenditure and body weight [25]. It is located on chromosome 18q21 in humans. Disruption of the *MC4R* gene leads to the obesity phenotype, which is related to T2DM [26]. Vaisse et al. claimed that rare heterozygous *MC4R* variants have been identified in obese children and adults in many populations [27]. Obesity is an important risk factor for the progression of T2DM [17].

Herein, we explored whether *MC4R* polymorphisms could contribute to T2DM risk in a Chinese Han population. In this study, we found that the mRNA level of *MC4R* was decreased in T2DM patients compared to healthy controls. However, the overall analysis revealed that the association between *MC4R* polymorphisms and T2DM risk was insignificant. Subsequently, we examined the correlation of *MC4R* polymorphisms and T2DM risk by stratification analysis. We found that rs17782313 in *MC4R* obviously reduced the susceptibility toT2DM among individuals younger than 60 years old. It has previously been demonstrated that the *MC4R*-rs17782313 polymorphism is strongly related to obesity in adults and children of European descent [28]. Moreover, Hardy et al. also demonstrated that rs17782313 was associated with weight and BMI. The association of this polymorphism with weight strengthened during childhood and adolescence, and weakened during adulthood [29]. This result suggested that the effect of *MC4R*-rs17782313 on disease risk was dependent on age. In addition, a study showed that rs12970134 increased the risk of T2DM among individuals of European descent [30], although this effect was not found in our study. In our analysis, rs6567160 reduced the susceptibility to T2DM among individuals ≤ 60 years old but was not associated with the clinical characteristics. However, Carvalho et al. suggested that rs6567160 was associated with a greater postpartum increase in HbA1c in women who had experienced gestational diabetes mellitus than in those who had not [31]. Additionally, rs663129 decreased the risk of T2DM among Han Chinese people. This finding was inconsistent with the discovery of Nikpay et al., which indicated that allele A of rs663129 increased the risk of both coronary artery disease and obesity in individuals of European ancestry [32]. The reason for these inconsistent results may be that the occurrence and development of T2DM are related to a variety of factors, including population, sample size, and environment. Together, these data highlighted the important role of *MC4R* polymorphisms in the occurrence of T2DM.

Moreover, these selected SNPs in the *PDX1* and *MC4R* genes can affect promoter histone marks, enhancer histone marks, DNAse, proteins bound, motifs changed, NHGRI/EBI GWAS hits, and GRASP QTL hits. Therefore, we presumed that these functions could modify the risk of T2DM by influencing gene expression. The specific mechanisms underlying these effects require further investigation.

There were several limitations in this study. First, this research was performed based on a Chinese Han population. Therefore, further research with subjects of different genetic backgrounds should be conducted to validate our results. Second, selection bias was an unavoidable problem in our research.

## Conclusions

In conclusion, our findings demonstrated that the variants in the *PDX1* and *MC4R* genes were related to susceptibility to T2DM in the Chinese Han population. These single polymorphic markers are considered to be new targets in the assessment and prevention of T2DM among Chinese Han people.

## Supplementary Information

**Additional file 1. Table S1**. Primer sequences of PDX1 and MC4R for PCR.** Table S2**. Basic information of candidate SNPs in the study.

## Data Availability

The datasets generated and/or analyzed during the current study are not publicly available due patient privacy but are available from the corresponding author on reasonable request.

## Abbreviations

DM: diabetes mellitus
T2DM: type 2 diabetes mellitus
*PDX1*: pancreatic and duodenal homeobox-1
*MC4R*: melanocortin receptor 4
HWEL: Hardy–Weinberg equilibrium
ORs: odds ratio
CI: 95% confidence intervals
