# Supplementary material for: PDX1 and MC4R genetic polymorphisms are associated with type 2 diabetes mellitus risk in the Chinese Han population
Source: BMC Med Genomics. 2021 Oct 25;14:249. doi: 10.1186/s12920-021-01037-3 (PMC8543917; doi:10.1186/s12920-021-01037-3)
Supplement: Supplementary file 1 — Additional file 1. Table S1. Primer sequences of PDX1 and MC4R for PCR. Table S2. Basic information of candidate SNPs in the study. [file 12920_2021_1037_MOESM1_ESM.docx]

**Additional file 1**

**Supplement table 1 Primer sequences of *PDX1* and *MC4R* for PCR**

| Target gene | Forward primer (from 5′ to 3′) | Reversed primer (from 5′ to 3′) |
| --- | --- | --- |
| *PDX1* | GAGCTGGAGAAGGAGTTCCTATTCAAC | TGATGTGTCTCTCGGTCAAGTTCAAC |
| *MC4R* | GAGCTGGAGAAGGAGTTCCTATTCAAC | TGATGTGTCTCTCGGTCAAGTTCAAC |
| GAPDH | GGAGCGAGATCCCTCCAAAAT | GGCTGTTGTCATACTTCTCATGG |

**Supplement table 2 Basic information of candidate SNPs in the study**

| SNP ID | Genes | Chr: Position | Alleles  Minor/major | MAF | | HWE  *p* | OR(95%CI) | *p* | HaploReg |
| --- | --- | --- | --- | --- | --- | --- | --- | --- | --- |
|  |  |  |  | Case | Control |  |  |  |  |
| rs11619319 | *PDX1* | 13:28487599 | G/A | 0.451 | 0.444 | 0.320 | 1.03(0.86-1.23) | 0.757 | Enhancer histone marks, DNAse, Proteins bound, Motifs changed |
| rs2293941 | *PDX1* | 13:28491198 | A/G | 0.446 | 0.440 | 0.414 | 1.02(0.86-1.22) | 0.791 | Promoter histone marks, Enhancer histone marks, DNAse, Motifs changed, NHGRI/EBI GWAS hits |
| rs9581943 | *PDX1* | 13:28493997 | A/G | 0.352 | 0.372 | 0.566 | 0.92(0.76-1.10) | 0.346 | Promoter histone marks, Enhancer histone marks, DNAse, Proteins Bound, Motifs changed, NHGRI/EBI GWAS hits |
| rs7981781 | *PDX1* | 13:28499962 | A/G | 0.434 | 0.431 | 0.523 | 1.01(0.85-1.21) | 0.897 | Promoter histone marks, Enhancer histone marks, DNAse, Motifs changed |
| rs6567160 | *MC4R* | 18:57829135 | C/T | 0.237 | 0.235 | 0.063 | 1.01(0.82-1.25) | 0.897 | Enhancer histone marks, DNAse, Motifs changed, NHGRI/EBI GWAS hits |
| rs663129 | *MC4R* | 18:57838401 | A/G | 0.238 | 0.234 | 0.061 | 1.03(0.83-1.26) | 0.814 | Enhancer histone marks, Motifs changed |
| rs17782313 | *MC4R* | 18:57851097 | C/T | 0.239 | 0.233 | 0.059 | 1.04(0.84-1.27) | 0.733 | Motifs changed, NHGRI/EBI GWAS hits, GRASP QTL hits |
| rs12969709 | *MC4R* | 18:57859563 | A/C | 0.219 | 0.230 | 0.100 | 0.94(0.76-1.16) | 0.572 | Enhancer histone marks, Motifs changed |
| rs11663816 | *MC4R* | 18:57876227 | C/T | 0.220 | 0.228 | 0.611 | 0.96(0.78-1.18) | 0.686 | Enhancer histone marks, Motifs changed |
| rs12970134 | *MC4R* | 18:57884750 | A/G | 0.206 | 0.217 | 0.511 | 0.94(0.76-1.17) | 0.578 | DNAse, Motifs changed, NHGRI/EBI GWAS hits, GRASP QTL hits |

SNP: Single nucleotide polymorphism; HWE: Hardy-Weinberg equilibrium; OR: Odds ratio; 95% CI: 95% confidence interval.
*P*-value obtained from Pearson's χ^2^ test.
